# Estimating Additive and Non-Additive Genetic Variances and Predicting Genetic Merits Using Genome-Wide Dense Single Nucleotide Polymorphism Markers

**DOI:** 10.1371/journal.pone.0045293

**Published:** 2012-09-13

**Authors:** Guosheng Su, Ole F. Christensen, Tage Ostersen, Mark Henryon, Mogens S. Lund

**Affiliations:** 1 Department of Molecular Biology and Genetics, Aarhus University, AU-Foulum, Tjele, Denmark; 2 Pig Research Centre, Danish Agricultural & Food Council, Copenhagen, Denmark; 3 School of Animal Biology, University of Western Australia, Crawley, Western Australia, Australia; University of Chicago, United States of America

## Abstract

Non-additive genetic variation is usually ignored when genome-wide markers are used to study the genetic architecture and genomic prediction of complex traits in human, wild life, model organisms or farm animals. However, non-additive genetic effects may have an important contribution to total genetic variation of complex traits. This study presented a genomic BLUP model including additive and non-additive genetic effects, in which additive and non-additive genetic relation matrices were constructed from information of genome-wide dense single nucleotide polymorphism (SNP) markers. In addition, this study for the first time proposed a method to construct dominance relationship matrix using SNP markers and demonstrated it in detail. The proposed model was implemented to investigate the amounts of additive genetic, dominance and epistatic variations, and assessed the accuracy and unbiasedness of genomic predictions for daily gain in pigs. In the analysis of daily gain, four linear models were used: 1) a simple additive genetic model (MA), 2) a model including both additive and additive by additive epistatic genetic effects (MAE), 3) a model including both additive and dominance genetic effects (MAD), and 4) a full model including all three genetic components (MAED). Estimates of narrow-sense heritability were 0.397, 0.373, 0.379 and 0.357 for models MA, MAE, MAD and MAED, respectively. Estimated dominance variance and additive by additive epistatic variance accounted for 5.6% and 9.5% of the total phenotypic variance, respectively. Based on model MAED, the estimate of broad-sense heritability was 0.506. Reliabilities of genomic predicted breeding values for the animals without performance records were 28.5%, 28.8%, 29.2% and 29.5% for models MA, MAE, MAD and MAED, respectively. In addition, models including non-additive genetic effects improved unbiasedness of genomic predictions.

## Introduction

Non-additive genetic variation results from interactions between genes. Interactions between genes at the same locus are called dominance, and interactions between genes at different loci are called epistasis. Although many studies have shown that non-additive effects have a substantial contribution to variation of complex traits [1]–[3], this source of variation is generally ignored in the genetic evaluation of complex traits.

Genome-wide dense single nucleotide polymorphism (SNP) markers have been widely used for association analysis [4]–[8] and genomic selection [9]–[12]. Unlike association analysis which aims at identifying quantitative trait loci (QTL) or chromosome regions with significant effect on the trait of interest, genomic selection focuses on predicting breeding values (total additive genetic effects). Similarly, when considering non-additive genetic effects, association analysis tries to find interactions among the specific genes that have a large effect on the trait of interest, while genomic selection pays attention to total non-additive genetic variations.

Many statistical models and algorithms have been proposed to predict breeding values using genome-wide dense markers, which differ in the assumption of distributions of SNP effects. Best linear unbiased prediction (BLUP) models [13]–[15] assume that effects of all SNP are normally distributed with equal variance. Variable selection models [12], [14], [16], [17] assume that marker effects have a thick-tailed distribution or a mixture distribution. Simulation studies with assumption that few QTL affect the trait of interest have shown that variable selection models are superior over BLUP models [14], [18], [19]. However, studies based on real dairy cattle and pig data indicate that BLUP models performed as well as variable selection models for most traits [11], [20], [21]. Therefore BLUP models have become popular approaches in practical genomic evaluations because they are simple and have low computational demands.

There are two BLUP models which have been widely used for genomic predictions. One estimates marker effects using random regression on marker genotypes and genomic breeding value of an individual is calculated as the sum of estimated marker effects (hereafter called as SNP-BLUP). The other estimates genomic breeding value directly using a marker-based relationship matrix (hereafter denoted as GBLUP). It has been shown that the GBLUP model is equivalent to the SNP-BLUP model [22], [23]. One of the advantages of the GBLUP model is that the model can use the framework of traditional pedigree-based BLUP models and is easy to use the information of non-genotyped animals through a combined relationship matrix [24]–[27].

Similar to traditional genetic evaluations, genomic predictions are usually carried out using a model that ignores non-additive effects. The hypothesis of this study is that statistical models which include additive and non-additive genetic effects will predict genetic merit more accurately and with less bias, when non-additive genetic effects have a substantial contribution to the genetic variation.

The objectives of this study were twofold. The first was to describe an approach to estimate additive and non-additive genetic variations and predict genetic values for complex traits using models integrating additive and non-additive genomic relationship matrices. The second was to estimate additive, epistatic and dominance genetic variances and access the accuracy of genomic predictions for daily gain in Danish Duroc pigs using models with or without including non-additive genetic effects.

## Materials and Methods

### Genomic BLUP model for additive and non-additive genetic effects

A linear mixed model including additive and non-additive genetic effects can be written as:
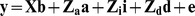
where **y** is the vector of observations, **b** is the vector of non-genetic effects, **a** is the vector of additive genetic effects, **i** is the vector of epistatic effects (second order epistasis in this study), **d** is the vector of dominance effects, and **e** is the vector of random residuals.

It is assumed that


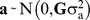
, 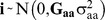, 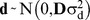, and 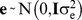,

where 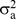
is the additive genetic variance, 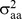 is the epistatic variance, 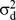 is the dominance variance, 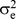 is the residual variance, **I** is an identity matrix, **G, G**_aa_ and **D** are the additive, epistatic and dominance genetic relationship matrices, respectively. These matrices can be constructed from either the information of pedigree or the information of genome-wide markers. The marker-based relationship matrices have the advantage to capture both the Mendelian segregation and the genetic links through unknown common ancestors, which are not available in the known pedigree. The present study will demonstrate the calculation of additive, dominance and epistatic genetic relationship matrices based on genome-wide markers. In the context, the three marker-based relationship matrices will be denoted as additive, epistatic and dominance genomic relationship matrices.

#### Additive genomic relationship matrix

The additive genomic relationship matrix **G** can be constructed using SNP marker information according to the previous studies [13], [15]. Briefly, 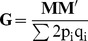, where **M** is a n×m matrix (n =  number of animals, m = number of marker loci) which specifies SNP genotype coefficients at each locus. The coefficients of the i^th^ column in the **M** matrix are (0 - 2p_i_) for genotype A_1_A_1_, (1-2p_i_) for A_1_A_2_, and (2-2p_i_) for A_2_A_2_, where q_i_ and p_i_ are the frequencies of allele 1 (A_1_) and allele 2 (A_2_) at locus i, respectively.

#### Epistatic genomic relationship matrix

According to the previous study [28], the epistatic genomic relationship matrix can be derived from additive genomic relationship matrix. When considering only second order epistasis (i.e., additive by additive interactions) and ignoring inbreeding, the epistatic genomic relationship matrix is: 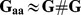, where # denotes the Hadamard product operation.

#### Dominance genomic relationship matrix

The dominance genomic relationship matrix **D** can be derived as follows. Denote u as the dominance value at a single locus and h_0_ as the heterozygosity of an individual at the locus. The dominance effect of an individual at a locus can be described as 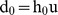. For a locus with two alleles (say A_1_ and A_2_), 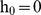
for the homozygous genotypes A_1_A_1_ and A_2_A_2_, and 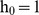
for the heterozygous genotype A_1_A_2_. Since h_0_ is either 0 or 1, 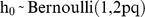. Therefore, 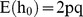
and 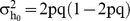.

To simplify the algorithm in the above linear mixed models, it is assumed that dominance effects (d) are normally distributed with mean equal to zero. In order to meet this assumption, dominance effects are expressed as deviations from the population mean, 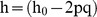. Thus, let 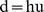, then 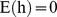, 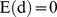, 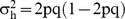
and 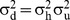, where 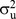 is the variance of dominance values.

Expanding to m loci, the genome-wide dominance effect of an individual is 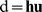, where **h** is the m-dimensional vector of heterozygosity coefficients, and **u** is the m-dimensional vector of dominance values. Assuming that dominance values at different loci are identically and independently distributed normal variables, the variance of genome-wide dominance effects in a Hardy-Weinberg equilibrium population is
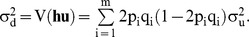



Expanding to n individuals and m loci,



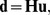
where **d** is the n-dimensional vector of dominance effects, **H** is the n×m matrix of heterozygosity coefficients with element 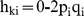 if individual k is homozygous, and 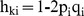
if individual k is heterozygous at locus i. The covariance structure of **d** is
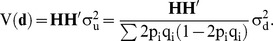



Consequently, the dominance genomic relationship matrix **D** is,
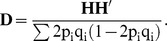



By centering h_ki_ with 2p_i_q_i_ and scaling **HH**' with 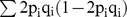, the **D** matrix has the properties that the expectation of an off-diagonal element is zero for two unrelated individuals, and the expectation of a diagonal element is one for a non-inbred individual. The **D** matrix is a realized dominance relationship matrix and is analogous to the pedigree-based numerator dominance relationship matrix.

### Marker data and phenotypic data of daily gain in pigs

#### Marker data

SNP marker data were obtained from 1911 Danish Duroc pigs (most were boars) which were genotyped using Illumina PorcineSNP60 BeadChip (Illumina, San Diego, CA). The SNP data were edited using the following criteria: 1) Minor allele frequency higher than 5%; 2) locus call rate larger than 0.95; and 3) individual animal call rate larger than 0.95. In addition, if the GenCall score of a single SNP in an animal was less than 0.65, the SNP in this animal was treated as missing and the corresponding genotype coefficient was replaced by the expected genotype coefficient at this locus. After editing, there were 26,142 SNP markers available for 1,911 pigs (born from 1996–2009, with 77% born during 2006–2009).

#### Phenotypic data

The analyzed trait was average daily gain (**DG**) from 30 kg to 100 kg. More details about the data can be found in the previous study [21]. Five datasets were used in the current analysis. DG of 339,393 individuals born during 1992–2009 (**DATA_all_**) were used to calculate corrected phenotypic values of DG (see detail below). The corrected DG of the 1,911 genotyped pigs (**DATA_gen_**) were used to estimate additive and non-additive genetic variances. The data were further divided into reference dataset (**DATA_ref_**, n = 1,484) and test dataset (**DATA_test_**, n = 427) by a cut-off date June 1, 2008 (birth date). DATA_ref_ were used to predict breeding value of the genotyped pigs in DATA_test_.

Corrected phenotypic values of daily gains (**DG_c_**), instead of original observations, were used as response variables to estimate additive and non-additive genetic variances and to predict genetic effects using SNP markers. The reason for using DG_c_ as response variables was to reduce noise by removing contemporary group effects which could be estimated much more accurately using a large dataset including all contemporaries and relatives, rather than using only genotyped animals. The contemporary group effects were estimated using a traditional pedigree-based linear model including sex, herd-week-section, pen, litter and additive genetic effects as well as random residuals. The DG_c_ was defined as original observations of daily gain adjusted for all non-genetic effects except for litter effect because based on this model it included part of non-additive genetic effects that should not be corrected for.

### Statistical analysis of daily gain

Four linear mixed models were used to estimate variance components and predict genetic effects, based on DG_c_ of genotyped animals.
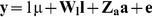
(MA)

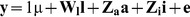
(MAE)

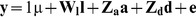
(MAD)

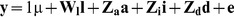
(MAED)where **y** is the vector of DG_c_, μ is the intercept, **l** is the vector of litter effects and is assumed that 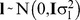, and the definitions of **a**, **i**, **d** and **e** are the same as stated above.

The analyses were carried out applying the average information restricted maximum likelihood algorithm [29] implemented in the DMU package (http://dmu.agrsci.dk).

### Model Validation

Goodness of fit for each model was evaluated by likelihood value based on the dataset DATA_gen_. The superiority of an alternative model over model MA was tested using a likelihood ratio test. The predictive ability of the model (with respect to accuracy and unbiasedness) was evaluated by comparing predictions and DG_c_ of animals in the test dataset (DATA_test_). Prediction accuracy was measured as the correlation between predicted genetic values and DG_c_. Both predictions of additive genetic effect (breeding value) and total genetic value (defined as the sum of the genetic effects in the model) were evaluated. Hotelling-Williams t-test [30], [31] was implemented to test if the correlations obtained from these prediction methods were significantly different. By definition, the reliability of predicted additive genetic effects is the squared correlation between predicted and true additive genetic effects, 
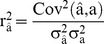
. Since true additive genetic effects were unknown, the reliability of predicted additive genetic effects was calculated as the squared correlation between predicted additive genetic effects and DG_c_, divided by heritability of DG_c_, i.e., 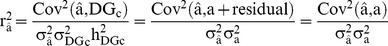. Here, the heritability 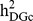
was the narrow-sense heritability estimated from the full model MAED. Unbiasedness of genomic predictions was measured using the regression of DG_c_ on the genomic predictions. A necessary condition for unbiased predictions is that the regression coefficient does not deviate significantly from one.

## Results

### Mean and standard deviation of daily gain

The mean and standard deviation of DG_c_ (the corrected daily gain) were significantly different in the different datasets (Table 1). This was due to continuous selection for this trait, which led to a continuous genetic progress. Animals with records in the whole dataset were born during the period from 1992 to 2009 (almost the same number of animals per year), while most genotyped animals were born during the period from 2006 to 2009. Thus, the mean DG_c_ in the whole dataset was lower than that in the dataset of genotyped animals. Similarly, the animals in the reference dataset were born before those in the test dataset and consequently had a lower mean in DG_c_. The selection also resulted in a larger overall standard deviation of DG_c_ in the dataset covering a longer period, because the standard deviation included the variation of year means. Therefore, the within-year standard deviation is a more appropriate measure of the variation of this trait. The coefficients of within-year variation for DG_c_ in different datasets ranged from 5.7% to 7.7%.

**Table 1:** Mean, total standard deviation (SD_t_) and within-year standard deviation (SD_w_) of the corrected phenotypic values of daily gain in different datasets.

Dataset	N	Mean	SD_t_	SD_w_
All	339,393	964	134	74
Genotyped	1,911	1,134	78	67
Reference	1,484	1,125	77	64
Test	427	1,164	74	74

### Estimates of variance components and heritability

The dominance variance accounted for 5.6% of the phenotypic variance (Table 2). The epistatic variance accounted for about 9.5% of the phenotypic variance, but the estimate had a large standard error, and thus was not statistically significantly different from zero. Model MA resulted in the largest estimates of additive genetic, litter and residual variances, while model MAED led to smallest estimates of these variances, and estimates from model MAE and MAD were intermediate in size. The results suggested that when a model excluded non-additive genetic effects, the variation due to non-additive genetic effects was distributed to other variance components in the model. Estimates of narrow-sense heritability (proportion of additive genetic variance to phenotypic variance) were 0.397, 0.373, 0.379 and 0.357 from model MA, MAE, MAD and MAED, respectively. Based on model MAED, the estimate of broad-sense heritability (proportion of the sum of additive, epistatic and dominance genetic variances to phenotypic variance) was 0.506.

**Table 2:** Estimates of additive genetic variance (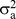), epistatic variance (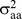), dominance variance (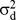), litter variance (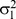), residual variance (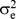) and their standard errors, and the proportions of these variances (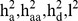) to phenotypic variance (defined as the sum of variance components in the model).

Parameters	MA	MAE	MAD	MAED
σ^2^_a_	2,176±241	2,029±255	2,081±246	1,942±260
σ^2^_aa_	-	529±429	-	506±429
σ^2^_d_	-	-	309±175	303±175
σ^2^_l_	604±231	523±237	542±231	465±238
σ^2^_e_	2,707±241	2,362±371	2,557±252	2,231±376
h^2^_a_	0.397**	0.373**	0.379**	0.357**
h^2^_aa_	-	0.098	-	0.093
h^2^_d_	-	-	0.056*	0.056*
l^2^	0.110*	0.096*	0.099*	0.085*

* : Significantly differ from 0 at P<0.05.

** : Significantly differ from 0 at P<0.01

### Goodness of fit

Measures of goodness of fit are given in Table 3. Model MA had the largest -2 log likelihood, followed by MAE and then by MAD, whereas model MAED had the lowest -2 log likelihood and thus fitted the data best. However, the likelihood ratio test showed that model MAE did not fit data significantly better than MA. On the other hand, model MAD and MAED were superior to model MA at a level approaching to statistical significance (P = 0.054 for MAD vs. MA, P = 0.067 for MAED vs. MA). These results indicated that the goodness of fit was improved by including dominance effects, but no detectable improvement was observed by including epistatic effects.

**Table 3:** -2log likelihood, χ^2^ value and the corresponding P-value of likelihood ratio.

Model	-2logL	?^2^-value^a)^	P-value
MA	18019.6		
MAE	18017.8	1.8	0.180
MAD	18015.9	3.7	0.054
MAED	18014.2	5.4	0.067

^a)^ 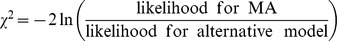

### Accuracy of prediction

Model MAED led to the highest correlation between predicted total genetic values and DG_c_, followed by MAD, then followed by MAE, and MA yielded the lowest correlation (Table 4). The same pattern was observed in correlations between predicted additive genetic effects (which is typically defined as breeding value) and DG_c_. Reliabilities of predicted additive genetic effects were 28.5%, 28.8%, 29.2% and 29.5% for models MA, MAE, MAD and MAED, respectively. The Hotelling-Williams t test showed that the difference in accuracies of predictions using these models were statistically significant from zero, except for predictions of total genetic values between models MA and MAE. The results indicated that including non-additive genetic effects in a prediction model improve accuracy of genomic predictions.

**Table 4:** Correlation between corrected daily gain (DG_c_) and predicted total genetic value (GTV, defined as the sum of genetic effects in the model), between DG_c_ and estimated additive genetic effect (GBV), and reliability of GBV (R^2^_GBV_), for the animals in the test dataset.

Model	Cor(GTV, DG_c_)	Cor(GBV, DG_c_)	R^2^_GBV_ (%)
MA	0.319^a^	0.319^a^	28.5^a^
MAE	0.320^a^	0.321^b^	28.8^b^
MAD	0.330^b^	0.323^c^	29.2^c^
MAED	0.331^c^	0.325^d^	29.5^d^

a-d: Within a column, estimates without a common superscript differ significantly (*P*<0.05), according to the Hotelling-Williams t test.

### Unbiasedness of prediction

As shown in Table 5, the range of regression coefficients of DG_c_ on predicted total genetic value was between 0.927 (model MA) and 0.985 (model MAED), the regression coefficients of DG_c_ on predicted additive genetic values ranged from 0.927 (model MA) to 1.029 (model MAED). For all models, the regression coefficients were not significantly different from 1, indicating the predictions were not significantly biased. However, the regression coefficients for the predictions using the models that included the non-additive genetic effects were closer to 1, suggesting that these models slightly improved the unbiasedness of genomic predictions.

**Table 5:** Regression coefficients (± standard errors) of corrected daily gain on predicted total genetic value (GTV) and on predicted breeding value (GBV) for the animals in the test dataset.

Model	Reg. on GTV	Reg. on GBV
MA	0.927±0.134	0.927±0.134
MAE	0.954±0.137	0.981±0.140
MAD	0.959±0.133	0.983±0.140
MAED	0.985±0.136	1.029±0.147

## Discussion

This study described a novel approach to model additive, epistatic and dominance genetic effect. The approach, by constructing additive and non-additive genetic relationship matrices using information of genome-wide SNP markers, is generally applicable for different populations with or without pedigree information. The method was used to investigate the variance components of additive and non-additive genetic effects and the accuracy of genomic predictions for DG in Danish Duroc population. The estimates of dominance variance and epistatic variance indicated that non-additive genetic effects had a substantial contribution to total genetic variation of this trait. The models including non-additive genetic effects predicted breeding values more accurately and unbiasedly, compared with a model ignoring non-additive genetic effects.

### Additive and non-additive genomic relationship matrices

Genome-wide dense markers provide a new approach to detect non-additive genetic variation and predict genetic merit. Additive genomic relationship matrix [13], [15] has been widely used for genomic prediction applying a linear mixed model [20], [32], [33]. The present study firstly demonstrated the method to construct dominance relationship matrix using genome-wide dense SNP markers. Following the previous study [28], epistatic genomic relationship matrix can be easily derived from additive genomic relationship matrix. The genomic relationship matrices represent the realized rather than expected sharing of ancestral genes. Compared to the pedigree-based relationship matrix, the genomic relationship matrix can capture both the Mendelian segregation and the genetic links through unknown common ancestors, which are not available in the known pedigree. Furthermore the genomic relationship matrices are applicable for different populations with or without pedigree information, which is particularly advantageous in studies on wild populations or human populations [34], [35].

The proposed method to construct epistatic genomic relationship matrix is an approximate approach and only second order epistasis (additive by additive interactions) is considered. Following previous report [28], higher order epistatic genomic relationship matrix can be approximately calculated as particular Hadamard product, such as **G**#**G**#**D** for additive by additive by dominance genomic relationship matrix, **G**#**G**#**G** for additive by additive by additive genomic relationship matrix. However, when higher order epistasis is involved in the analysis, more data information is required. A precise epistatic relationship matrix is difficult to be obtained when considering a large number of markers. Epistasis among a few specific markers can be modeled in a desired way [36]–[38]. However, instead of dealing with a small proportion of non-additive genetic variation from a few specific markers, the current study tries to use a feasible approach to account for overall non-additive genetic variation as accurate as possible.

With additive and non-additive genetic relationship matrices, additive and non-additive genetic variances and genetic values can be easily estimated using a typical linear mixed model, such as a GBLUP model. A GBLUP model is equivalent to a linear random regression model assuming that effects of all SNP are normally distributed with equal variance. A GBLUP model may be not satisfactory in the situation that few markers have large effect whole the most markers have null effect or very small effect. However, experiences with real dairy cattle data and pig data indicate that a BLUP model performed well for most traits. Therefore, the approach proposed in this study could be not an optimal but a simple and feasible approach for estimating additive and non-additive variances and predicting genomic breeding values.

### Additive and non-additive genetic variances of daily gain

Based on the present data, the estimated epistatic variance and dominance variance in proportion to additive genetic variance were about 26% and 15%, respectively. The estimates are in the range of those for complex traits reported in previous studies. Additive and non-additive variances are usually estimated using a model with a pedigree-based relationship matrix. In dairy cattle, it was reported that the ratios of dominance variance to additive genetic variance were 118% and 161%, and the ratios of epistatic variance to additive genetic variance were 59% and 2% for days open and service period, respectively, in US Holstein [39]; the ratio of dominance variance to additive genetic variance was 17% for stature in US Holstein [40]; and non-additive variances were, in general, as large as or greater than the additive variances for reproductive traits in Canadian Holstein [2]. In beef cattle, the ratio of dominance variance to additive genetic variance was larger than 50% for weaning weight in Hereford, Gelbvieh and Charolais beef cattle [1], [41], and for post-weaning gain in Limousin beef cattle [42]. In pigs, significant contributions of non-additive genetic variance have been reported. The ratios of dominance variance to additive genetic variance ranged from 11% to 31% for reproductive and growth traits in Yorkshire pigs [43]. The ratios were 15% for 21-day litter weight, 44% for number born alive, and 57% for interval between parities in South African Duroc pigs [3]. These results indicate non-additive genetic variations are important for complex traits, especially for fitness traits.

Several previous studies have reported non-additive genetic effects of detected QTL. In chicken, QTL analysis revealed that the non-additive genetic effect was more pronounced prior to 46 days of age, whereas additive genetic effect explained the major portion of the genetic variance later in life [44], [45]. In pigs, it was reported that the proportion of non-additive variance relative to the entire QTL variance exceeded 50% in most meat quality and carcass composition traits in a porcine Duroc × Pietrain population [46]. Rather than using detected QTLs, the present study estimated additive and non-additive genetic variances using genome-wide SNP markers. This is the first scientific report on estimation of on non-additive genetic variances in livestock using such an approach.

It has been observed that there is a large variation between the estimates of non-additive genetic variances in different studies, which may reflect the different features of various traits and populations. In addition, the large variation could be caused by large sampling error due to insufficient data information. As shown in this study, the standard errors of non-additive genetic variance were large. In fact, the estimated epistatic variance was not statistically significantly different from zero. The relative standard errors for the estimated dominance variance and epistatic variance (here defined as standard error/estimated variance) were 4.5 times and 6.5 times as large as that for additive genetic variance. Similarly, the relative standard error for dominance variance was 4.1 as large as that for additive genetic variance of stature in US Holstein [40]. These results suggest that a large dataset is needed in order to get accurate estimates of non-additive genetic variances.

### Genomic prediction of daily gain

The current and previous studies have shown that non-additive genetic variance is considerable in complex traits. Therefore, it is expected that a model including non-additive genetic effect would increase the prediction accuracy and improve the unbiasedness. In terms of practical animal breeding, focus is the prediction of breeding value (additive genetic value) or the genetic improvement in next generation. In this study, reliabilities of genomic predicted breeding value using model MAE, MAD and MAED were 0.3%, 0.7% and 1.0% units higher than that using the additive genetic model. In addition the models including non-additive genetic effects slightly improved unbiasedness of genomic prediction.

Compared to large non-additive genetic variances, the gain in reliability of genomic predictions by including non-additive genetic effects in the prediction model was relatively small. The small improvement indicates the difficulty to distinguish additive and non-additive genetic effects. Using traditional BLUP model with pedigree-based relationship matrix, a previous study on stature in US Holsteins [47] found that animals with large amount of additive information were influenced little by the inclusion of dominance, but large for the animals with a large amount of dominance information. The superiority of model MAE over model MA for genomic prediction is negligible, which was consistent with the large standard error for the estimates of epistatic variance. It should be noted that the current study was based on a small dataset. The data information might not be sufficient to distinguish additive genetic and non-additive genetic effects efficiently. It is expected that the benefit for genomic prediction by including non-additive genetic effects in the model will be larger when using larger reference dataset, especially when reference dataset including records of crossbred animals. Moreover, an additive and non-additive genetic model could be benefit for exploiting specific combining ability. A simulation study with various scenarios by [48] reported that the ratio of dominance response to additive genetic selection ranged from 3.8 to 16.6% by one generation of selection.

In a genetic evaluation system, there are two limitations in practice for using a model with both additive and non-additive genetic effects for genomic prediction. The first limitation is that the computational demand for models with both additive and non-additive genetic effects are generally high, since both additive and non-additive genomic relationship matrices are dense. This requires more powerful computers and/or more efficient algorithms. Second, a reference population often consists of sires which have records of progeny performance, and popularly used response variables are conventional estimated breeding value (EBV), de-regressed EBV or mean of corrected progenies' performances which are more informative than individual observation. However, these pseudo observations are appropriate for an additive genetic model, but not appropriate for a model that includes non-additive genetics effects because the interaction effects observed in the offspring are not related to interactions of genes in the sire. A genomic prediction model including non-additive genetic effects requires that the response variable is individual record. An extension of single-step model [24]–[26] to handle non-additive genetic effects may be a good approach, because this approach allows predicting breeding value using the observations from both genotyped and non-genotyped animals by combining genomic and pedigree information into a joint relationship matrix.

The current study was based on data from purebred Duroc pigs. Non-additive genetic variation in a crossbred population is expected to be much larger than that in a purebred population. In farm animals such like poultry, pig, and beef cattle, crossbreds are usually the end product. Nonetheless, selection is carried out in the purebreds to improve performance of the crossbreds. The information from crossbred population will be useful for increasing accuracy of genetic evaluation of animals in the purebreds due to additional information of the relatives in crossbred. Moreover, the information of crossbred population allows selecting candidates in purebreds for the performance of their crossbred offspring. According to the results from the present study, when using data of crossbreds, a model including non-additive genetic effects, especially dominance effect, is expected to yield a substantial improvement of genetic evaluations.

It can be concluded that the present method is a feasible approach to estimate additive and non-additive variances and predict genomic breeding values. Non-additive genetic effects are important sources of genetic variation for daily gain in pigs. Genomic prediction models including non-additive genetic effects can improve accuracy and unbiasedness of genomic predicted breeding value.
